# Single‐Cell Characterization of SPP1‐Expressing Macrophages and Their Predicted Interactions With T Cells in the Breast Cancer Bone Metastatic Microenvironment

**DOI:** 10.1002/cnr2.70732

**Published:** 2026-11-01

**Authors:** Zixue Xuan, Zhongxiu Wu, Yuting Zhong, Jinying Jiang, Yuan Zhang, Lei Cheng, Jun Zhang, Kai Wang

**Affiliations:** ^1^ Department of Pharmacy Zhejiang Provincial People's Hospital Bijie Hospital (The First People's Hospital of Bijie) Bijie Guizhou China; ^2^ Center for Clinical Pharmacy, Cancer Center, Department of Pharmacy Zhejiang Provincial People's Hospital (Affiliated People's Hospital), Hangzhou Medical College Hangzhou Zhejiang China; ^3^ Hangzhou Medical College Hangzhou Zhejiang China; ^4^ Department of Orthopedics Zhejiang Provincial People's Hospital Bijie Hospital (The First People's Hospital of Bijie) Bijie Guizhou China; ^5^ Center for Plastic & Reconstructive Surgery, Department of Orthopedics Zhejiang Provincial People's Hospital (Affiliated People's Hospital), Hangzhou Medical College Hangzhou Zhejiang China; ^6^ Key Laboratory of Epigenetics and Oncology, Research Center for Preclinical Medicine Southwest Medical University Luzhou China

**Keywords:** bone metastasis, breast cancer, immune microenvironment, Mac‐SPP1

## Abstract

**Background:**

Breast cancer is a highly common malignancy in women, with bone as its most frequent site of distant metastasis significantly increasing mortality risk.

**Methods:**

This study integrated single‐cell RNA sequencing datasets (GSE266330 and GSE243526) to systematically characterize the tumor microenvironment across normal tissue, primary tumor, and bone metastasis in breast cancer.

**Results:**

In total, 141 056 high‐quality single cells were analyzed from an integrated cohort comprising a bone‐metastasis dataset (GSE266330) and a primary/normal breast dataset (GSE243526), identifying 11 major cell types. Within the bone‐metastatic cohort, we identified a macrophage subpopulation highly expressing SPP1 (Mac‐SPP1) that exhibited an M2‐polarized signature and elevated metabolic gene‐set enrichment, and that expressed transcription factors such as HOXA9 and MAF. We also identified CD4‐S100A9 and CD8‐GZMK T‐cell subpopulations with high cytotoxicity, high exhaustion, and metabolic reprogramming. Pseudotime analysis positioned CD4‐S100A9 cells at one end of the inferred CD4+ T‐cell trajectory and CD8‐GZMK cells at an intermediate node along the inferred CD8+ T‐cell trajectory. Cell–cell communication analysis predicted that Mac‐SPP1 interacts with these T‐cell subsets through the SPP1‐integrin (e.g., SPP1‐CD44) and CD74‐MIF/COPA signaling axes.

**Conclusion:**

This study provides a single‐cell characterization of the breast cancer bone‐metastatic microenvironment, identifying an SPP1‐expressing macrophage subset (Mac‐SPP1) and predicting its interactions with CD4‐S100A9 and CD8‐GZMK T cells. Because the bone‐metastatic and primary/normal specimens derive from different datasets, these findings are descriptive and cannot be attributed to metastatic progression alone and require experimental validation and confirmation in cohorts that profile primary and metastatic specimens within a single study.

**Abbreviations**
LTβ: lymphotoxin‐β
Metastatic: bone metastases of breast cancer tissues
Normal: normal breast tissues
Primary: primary breast cancer tissues
scRNA‐seq: single‐cell RNA sequencing
TF: transcription factor

## 1 Introduction

Breast cancer is a highly common malignancy in women globally, holding the highest incidence rate and ranking as the fifth most common cause of cancer mortality worldwide [1]. Bone is the most common site of metastasis in breast cancer [2], and the occurrence of bone metastasis in breast cancer significantly increases mortality risk, highlighting the urgent need for focused research and intervention in the treatment of breast cancer [3]. Numerous studies have revealed that the tumor microenvironment, particularly specific cell subpopulations, plays a pivotal role in breast cancer bone metastasis. For instance, tumor‐derived granulocyte colony‐stimulating factor modulates the bone marrow vascular microenvironment [4]; proline dehydrogenase 2 influences hydroxyproline metabolism and promotes SLC7A11 and CXCL8 expression [5]; the 5‐methylcytosine methyltransferase NSUN2 accelerates the degradation of lysine demethylase 6B mRNA, thereby activating the Notch signaling pathway and increasing RANKL expression [6]; and the N6‐methyladenosine reader YTHDF3 enhances the translation of ZEB1 and SMAD5 [7]. All these mechanisms help promote breast cancer bone metastasis, illustrating the high complexity of the mechanisms driving breast‐cancer bone metastasis. Thus, further studies are still needed to elucidate such mechanisms.

Advances in single‐cell RNA sequencing (scRNA‐seq) technology have enabled accurate and in‐depth studies of the tumor microenvironment and cellular heterogeneity [8], thereby uncovering the mechanisms underlying breast cancer bone metastasis. Kaur J et al. identified transcriptomic alterations in senescent osteocytes in a mouse model of breast cancer bone metastasis, and another study showed that SCUBE2 is enriched in osteoblastic subpopulations of the early bone‐metastatic niche, suggesting a potential role in driving “seed‐and‐soil” interactions [9]. Wang et al. further showed that lymphotoxin‐β (LTβ) is highly expressed by tumor cells in the bone microenvironment, fostering their colonization and growth [10]. Complementing these findings, a recent study mapped the single‐cell landscape of primary breast tumors and adjacent normal tissue [11], while a second, large‐scale atlas profiled 42 bone metastases across eight solid‐tumor types, providing the first cross‐cancer comparison of shared and tumor‐specific bone‐metastatic programs, with breast cancer samples being a major component [12].

This study integrates two public single‐cell RNA‐sequencing datasets (GSE266330, bone metastases; GSE243526, primary tumors and normal breast tissue) to characterize the cellular heterogeneity of the breast cancer bone‐metastatic microenvironment, identify macrophage and T‐cell subpopulations present in the metastatic niche, and infer candidate molecular programs and cell–cell interactions for future investigation.

## 2 Methods

### 2.1 scRNA‐Seq Data Sources

scRNA‐seq data were obtained from GSE266330 [12] and GSE243526 [11], which were downloaded from the Gene Expression Omnibus (GEO) database (https://www.ncbi.nlm.nih.gov/geo/). The patient information used in this study is presented in Table S1. The transcriptome and clinical data of breast cancer were retrieved from The Cancer Genome Atlas Breast Invasive Carcinoma (TCGA‐BRCA) cohort (https://www.cancer.gov/tcga) [13]. Pathway enrichment analysis was performed using the Kyoto Encyclopedia of Genes and Genomes (KEGG) database (https://www.kegg.jp/kegg/pathway) [14]. Ligand‐receptor interaction analysis was conducted using CellPhoneDB v5.0 (https://www.cellphonedb.org) [15].

### 2.2 Analysis of scRNA‐Seq Data

2.2.1

Raw count matrices were subjected to stringent quality control before downstream analysis. Cells with fewer than 200 or more than 7000 detected genes (nFeature_RNA) were removed to exclude empty droplets and suspected doublets, respectively. Cells with more than 50 000 total transcript molecules (nCount_RNA) were additionally excluded as likely doublets. Cells with a mitochondrial gene percentage (percent.mt) exceeding 20% were removed to filter out low‐quality or dying cells. scRNA‐seq data was analyzed using the Seurat package (version 5.0.3) in R software (version 4.2.2). We first performed batch integration using the FindIntegrationAnchors and IntegrateData functions, selecting the top 30 principal components (dims = 1:30). To remove batch effects, we applied the Harmony tool for data correction [16]. We selected the top 21 Harmony features for cell clustering with the FindClusters function at a resolution of 0.5. For both myeloid and T cell subpopulations, clustering was performed after dimensionality reduction and batch correction. Briefly, after quality control and normalization using the LogNormalize method (scale factor 10 000), the top 2000 variable features were identified using the FindVariableFeatures function for each subset. Batch effects were corrected using Harmony. For myeloid subpopulations, the first 12 Harmony components were used for clustering with the FindClusters function (resolution = 0.5), whereas for T cell subpopulations, the first 23 Harmony components were used (resolution = 0.5). Cluster identities were visualized using Uniform Manifold Approximation and Projection (UMAP) plots generated from the respective Harmony embeddings. Major cell types were annotated using canonical marker genes, with reference to the source‐dataset publications and established breast‐cancer single‐cell atlases: Fibroblasts were distinguished by COL1A1, COL1A2, and DCN; perivascular‐like cells by RGS5; cycling cells were defined by G2M/S‐phase gene scoring (Seurat's CellCycleScoring with canonical S‐ and G2M‐phase markers); and the HSPA6 cluster was interpreted as a heat‐shock‐induced stress state. Cell‐type proportion differences between groups were analyzed at the sample level using the Wilcoxon rank‐sum test, with *p* < 0.05 considered statistically significant. In addition, KEGG enrichment analyses were performed for each subtype feature gene using the R package clusterProfiler (v4.6.2) [17, 18].

### 2.3 Pseudotime Trajectory Inference Based on Monocle 2

We performed pseudotime trajectory analysis of the cells using the R package Monocle 2. The dpFeature function was used for unsupervised cell ordering and for selecting ordering genes. Subsequently, the cells were arranged along the trajectory through dimensionality reduction using the DDRTree algorithm.

### 2.4 M1/M2 Signature Score Analysis

M1/M2 polarization scoring was performed using the singscore algorithm for single‐sample gene set scoring. The polarization score for each cell was calculated based on literature‐reported M1‐type (IL1B, CCL2, CCL3, CCL4, TNF, CXCL2, CXCL9, CXCL10, and SOCS3) and M2‐type (CD163, MRC1, MSR1, CCL13, CCL18, IL10, FOLR2, STAB1, CD163L1, SEPP1, F13A1, MERTK, AXL, CCL22, IL1RN, GPNMB, and CHI3L1) signature genes [19]. For inter‐group comparisons, the Wilcoxon rank‐sum test was used according to the experimental design. Statistical significance was set at adjusted *p* < 0.05.

### 2.5 T Cell Cytotoxicity/Exhaustion Status Assessment

Cytotoxicity (GZMA, GZMB, GZMH, GZMK, GZMM, GNLY, PRF1, FASLG, IFNG, TNF, IL2R, and IL2) and exhaustion (CXCL13, HAVCR2, PDCD1, TIGIT, LAG3, CTLA4, LAYN, RBPJ, VCAM1, GZMB, TOX, and MYO7A) gene signatures were defined based on previous studies [20, 21]. Cytotoxicity and exhaustion scores for each T cell were calculated using the singscore algorithm. Differences between groups were analyzed using the Wilcoxon rank‐sum test.

### 2.6 Survival Analysis

To evaluate the prognostic relevance of the Mac‐SPP1 macrophage subset in bulk tumor data, we defined a five‐gene Mac‐SPP1 signature comprising two pan‐macrophage markers (LYZ, TYROBP) and three Mac‐SPP1‐specific markers (SPP1, FRMD4A, FCGBP), selected from the Mac‐SPP1 cluster marker genes identified in our scRNA‐seq analysis. Bulk RNA‐seq data (RNA Seq V2 RSEM) and clinical annotations for the TCGA‐BRCA cohort (PanCancer Atlas 2018; *n* = 1082 primary tumors, 151 deaths) were retrieved from cBioPortal. For each patient, the Mac‐SPP1 score was computed as the log2‐transformed mean of the five gene RSEM values, that is, score = log2(mean(RSEM_LYZ, RSEM_TYROBP, RSEM_SPP1, RSEM_FRMD4A, RSEM_FCGBP) + 1). The association with overall survival was assessed using a Cox proportional‐hazards model with the score as a continuous predictor, a model additionally adjusted for age, pathologic stage, and PAM50 subtype, and a median‐split Kaplan–Meier analysis with the log‐rank test.

### 2.7 Transcription Factor (TF) Analysis

PySCENIC is a Python implementation of the SCENIC (Single Cell Regulatory Network Inference and Clustering) workflow, which reconstructs transcriptional regulatory networks, identifies key TFs, and characterizes cell identities from scRNA‐seq data [22, 23]. We randomly selected 500 cells per group and performed TF analysis on different subpopulations using pySCENIC (v0.12.1).

### 2.8 Metabolic Gene‐Set Enrichment Analysis

To assess the metabolic transcriptional state of each cell subtype, we performed single‐cell enrichment analysis using the singscore algorithm with KEGG metabolic pathway gene sets as reference signatures. Enrichment scores were calculated for each cell individually, and the average score per subtype or per sample group was calculated to compare metabolic gene‐set enrichment across biological conditions. We note that these scores reflect enrichment of metabolic gene‐set expression and do not directly measure metabolic flux or metabolic activity.

### 2.9 Cell–Cell Communication Analysis

To infer potential ligand–receptor relationships between the Mac‐SPP1 macrophage subset and the CD4‐S100A9 or CD8‐GZMK T‐cell subsets, we performed a cell–cell communication analysis using CellPhoneDB v5.0.0 with default statistical‐analysis parameters (gene expression threshold of 10%, 1000 permutations, *p* < 0.05). Cells of each cell type were pooled across all biological samples for the analysis; because this pooled approach does not account for between‐sample biological replication, the results are interpreted as population‐level predicted ligand–receptor relationships. Because CellPhoneDB infers communication from the co‐expression of ligand–receptor pairs, the resulting interactions represent predicted signaling relationships and do not establish in vivo signaling or causal induction of T‐cell exhaustion [24]. Osteoclasts and osteoblasts were not distinguished as separate clusters in the integrated dataset and were therefore not included in the communication analysis. The complete CellPhoneDB output is provided in Tables S11 and S12.

### 2.10 Statistical Analysis

Proportion comparisons among the Normal, Primary, and Metastatic groups were performed at the sample level using the Wilcoxon rank‐sum test, with each specimen contributing one observation (*n* = 12, 8, and 4, respectively). Signature scores (M1/M2, cytotoxicity, exhaustion, and metabolic enrichment) were computed per cell using the singscore algorithm and compared across groups using the Wilcoxon rank‐sum test; because these cell‐level comparisons may be affected by within‐patient correlation, we additionally aggregated per‐cell scores to the sample level (mean score per specimen per cell type) and repeated the comparisons at the sample level. Multiple testing was controlled using the Benjamini–Hochberg method. Statistical significance was set at adjusted *p* < 0.05 (Benjamini–Hochberg).

## 3 Results

### 3.1 Tumor Microenvironment in Breast Cancer Bone Metastasis Characterized by Single‐Cell Transcriptomic Sequencing

We employed scRNA‐seq to systematically characterize the immune microenvironment across three tissue compartments, normal breast tissue (Normal), primary breast cancer tissue (Primary), and the bone‐metastatic niche (Metastatic). Because publicly available single‐cell data for breast‐cancer bone metastasis are limited, and matched normal‐primary‐metastasis specimens from the same patient are rarely available, the three compartments were profiled by integrating two public datasets (GSE266330 and GSE243526). We emphasize that this is a cross‐sectional comparison across tissue compartments rather than a longitudinal trajectory, and that the metastatic and primary samples are not patient‐matched; observed differences therefore reflect a combination of disease stage, tissue environment, and inter‐individual variation. Following strict quality control and doublet removal, 141 056 cells were retained for subsequent analyses. Integration and unsupervised clustering analysis was performed for all samples. As shown in Figure 1A, the UMAP plot evidently shows 11 cell populations, namely T cells, fibroblasts, epithelial, myeloid cells, perivascular‐like, endothelial, mast, B cells, HSPA6, cycling, and plasma (Tables S2 and S3). Subsequently, we analyzed the distribution of cell populations across different groups. The results revealed that compared with the Normal and Primary groups, the Metastatic group exhibited significant changes (Figure 1B). The marker genes of different cell populations are shown in Figure 1C. The bar plots in Figure 1D,E illustrate the proportional distribution of various cell types across different groups (Table S4). Some cells, such as Myeloid cells, T cells, and fibroblasts, exhibited notable differences. Further statistical analysis revealed a significant increase in the proportion of Myeloid cells and T cells in the Primary groups compared with the Normal group (Figure 1F), and a significant increase in the proportion of B cells, cycling, plasma, and T cells in the Metastatic groups compared with the Normal group (Figure 1G). In addition, the proportion of myeloid cells was significantly higher in the Metastatic group than in the Normal and Primary groups (Figure 1H). These findings indicate that myeloid and T‐cell compartments are abundant in the bone‐metastatic cohort. Because the metastatic and primary/normal specimens derive from different datasets, however, these differences may reflect dataset and tissue‐environment effects in addition to any biology of metastasis. These differences were also large in magnitude: the median myeloid‐cell proportion rose from 0.58% in the Normal group to 29.6% in the Metastatic group (Cliff's delta = −0.98 [95% CI: −1.00, −0.88], adjusted *p* = 3.2e‐05, Primary vs. Metastatic), and the median T‐cell proportion from 2.1% (Normal) to 17.6% (Metastatic) (Cliff's delta = −1.00, adjusted *p* = 1.1e‐03, Normal vs. Metastatic). Effect sizes and 95% confidence intervals for all cell‐type proportion comparisons are provided in Table S13.

**FIGURE 1:**
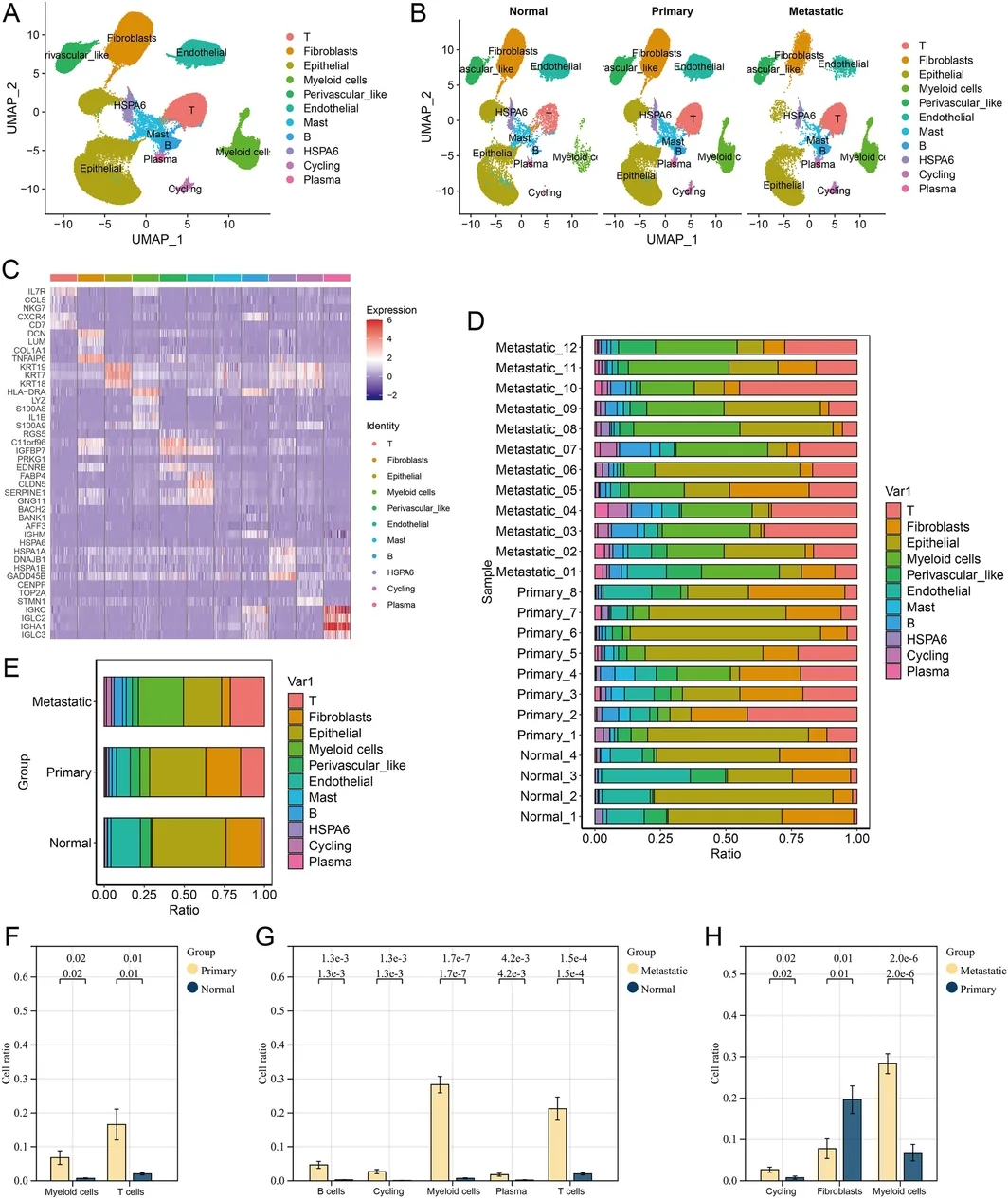
Single‐cell transcriptomic analysis of Normal, Primary, and Metastatic groups. (A) UMAP visualization of single cells colored by cell type, with shading indicating clusters within each type. (B) UMAP plots of single cells from the Normal, Primary, and Metastatic groups. (C) Heatmap showing expression levels of marker genes across different cell populations. (D, E) Proportions of each cell type among total cells per group. (F–H) Comparison of cell‐type proportions between groups: (F) Normal versus Primary, (G) Normal versus Metastatic, and (H) Primary versus Metastatic. Statistical significance was assessed using the two‐sided Wilcoxon rank‐sum test.

### 3.2 Characteristics and Functional States of Myeloid Cells in the Breast Cancer Bone Metastatic Microenvironment

Nine cell subpopulations were clustered and annotated based on marker genes, namely Mono‐S100A8, Mac‐SPP1, Mac‐CXCL3, Mac‐APOE, Mono‐FCGR3A, Mono‐GPX1, cDC, DC‐LAMP3, and Cycling Myeloid (Figure 2A,B). The marker gene expression patterns of these nine cell myeloid subpopulations are shown in a heatmap (Table S5, and Figure 2C). Of note, the Mac‐SPP1 subpopulation was composed almost entirely of cells from the bone‐metastatic dataset (2132 of 2135 cells, 99.9%), whereas Mac‐CXCL3 (2010 of 2010 cells) and Mac‐APOE (1625 of 1822 cells, 89.2%) were predominantly derived from the primary/normal dataset; the apparent expansion of Mac‐SPP1 in the metastatic group and of Mac‐CXCL3/Mac‐APOE in the primary group is therefore fully confounded with source dataset (Table S6, Figure 2D,E), and we interpret these as dataset‐associated clusters rather than metastasis‐specific cell states. To infer the macrophage differentiation course, we used Monocle 2 to perform trajectory analysis of these three cell subpopulations. As shown in Figure 2F, the inferred trajectory positioned the Mac‐SPP1 subpopulation at a terminal end with the highest pseudotime score, adjacent to the Mac‐APOE and Mac‐CXCL3 states; we note that this represents an inferred transcriptional ordering rather than demonstrated lineage ancestry. To investigate the functional properties of key macrophage subpopulations, we assessed their M1 and M2 polarization gene signatures (Table S7). The Mac‐SPP1 subpopulation exhibited an M2‐polarized signature (Figure 2G). Correspondingly, the overall macrophage population in metastases also showed a significant bias toward the M2 state (Figure 2H). To define the specific functional program of Mac‐SPP1 macrophages, we profiled genes associated with immune suppression (TGFB1, IL10), angiogenesis (VEGFA), and matrix remodeling (MMP9, MMP12). Relative to the other macrophage subsets, Mac‐SPP1 cells exhibited higher expression of TGFB1, VEGFA, SPP1, MSR1, and TREM2, whereas IL10, MMP9, and MMP12 were not elevated (Figure 2I). Because Mac‐SPP1 cells derive almost exclusively from the bone‐metastatic dataset, within‐subset primary‐versus‐metastatic comparisons were not feasible; within the bone‐metastatic cohort, MMP9 was among the matrix‐remodeling genes expressed by Mac‐SPP1 cells (Figure 2J). KEGG pathway enrichment analysis revealed that differentially expressed genes (DEGs) in macrophages from the Primary and Metastatic groups were primarily enriched in pathways such as osteoclast differentiation (Figure 3A).

**FIGURE 2:**
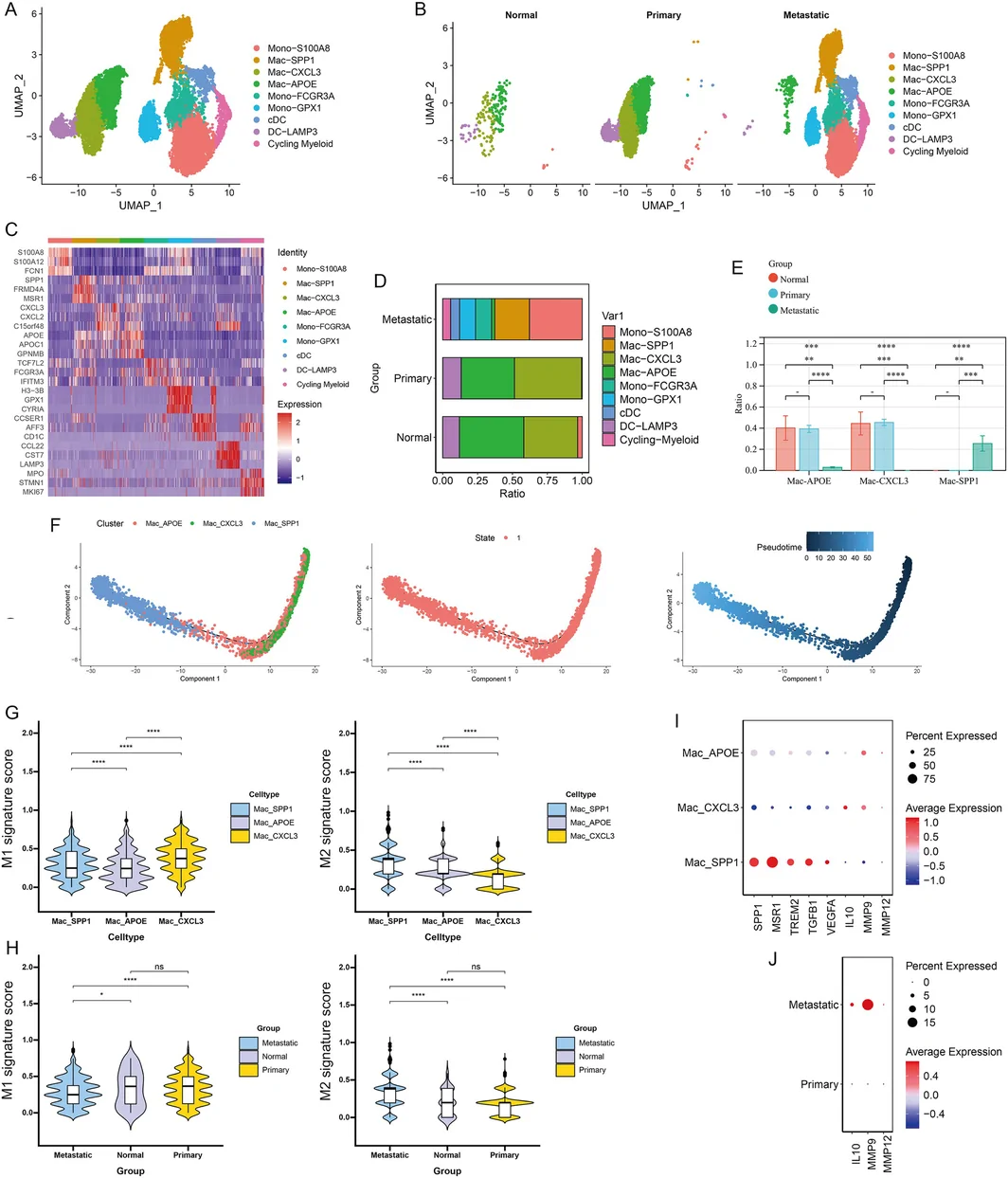
Transcriptional and cellular landscape of myeloid cells. (A) UMAP visualization of myeloid cell subpopulations. (B) UMAP plot showing the distribution of myeloid cells grouped by sample sites, with cells color‐coded into nine clusters. (C) Heatmap showing expression levels of marker genes across different myeloid subpopulations. (D, E) Proportions of each myeloid subpopulation among total myeloid cells per group. Statistical significance was assessed using the two‐sided Wilcoxon rank‐sum test. (F) Pseudotime trajectory of differentiation of macrophage subpopulations (Mac‐APOE, Mac‐CXCL3, and Mac‐SPP1) inferred by Monocle. (G, H) M1/M2 polarization scores were computed per cell with the singscore algorithm from curated gene sets—M1 (IL1B, CCL2, CCL3, CCL4, TNF, CXCL2, CXCL9, CXCL10, SOCS3) and M2 (CD163, MRC1, MSR1, CCL13, CCL18, IL10, FOLR2, STAB1, CD163L1, SEPP1, F13A1, MERTK, AXL, CCL22, IL1RN, GPNMB, CHI3L1). (I) Dot plots showing expression of the immunosuppressive factor TGFB1, the pro‐angiogenic factor VEGFA, and the markers SPP1, MSR1, and TREM2 across macrophage subpopulations (Mac‐SPP1, Mac‐APOE, Mac‐CXCL3). (J) Dot plot showing the expression of immune‐suppressive (IL10) and matrix‐remodeling (MMP9, MMP12) genes in Mac‐SPP1 macrophages from primary and metastatic sites.

**FIGURE 3:**
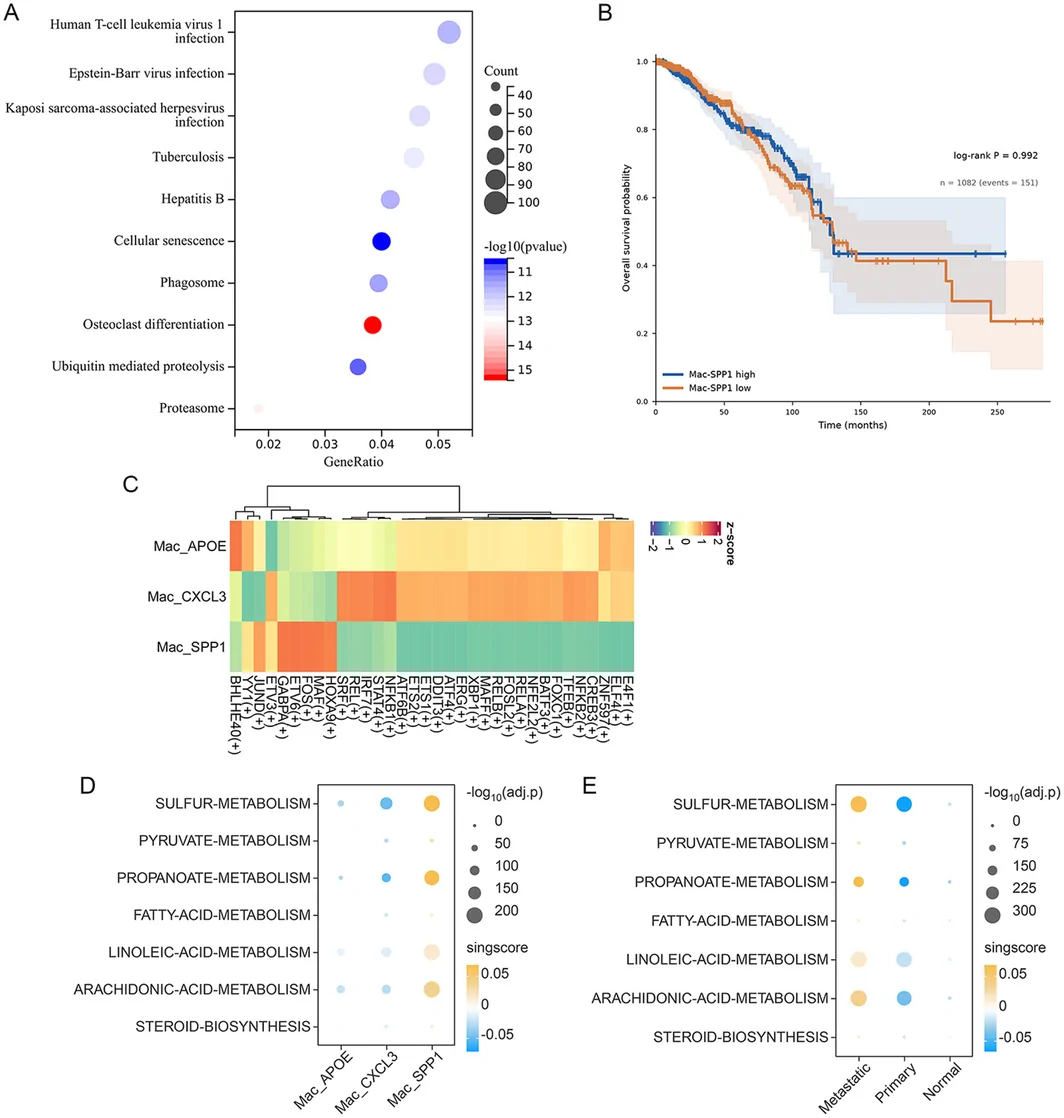
Mac‐SPP1 macrophage subpopulation: Survival association, TF activity, and metabolic gene‐set enrichment. (A) KEGG pathway enrichment analysis of DEGs in macrophages from the Primary and Metastatic groups. (B) Kaplan–Meier curves of overall survival in the TCGA‐BRCA cohort (*n* = 1082) stratified by the Mac‐SPP1 signature (median split); *p* value from the log‐rank test. (C) Heatmap of TF activity signatures across macrophage subpopulations. (D) Metabolic gene‐set enrichment score of the three macrophage cell subtypes. (E) Metabolic gene‐set enrichment score across the Normal, Primary, and Metastatic groups.

### 3.3 Transcriptional and Metabolic Characterization of Mac‐SPP1 Macrophages

We next evaluated whether the Mac‐SPP1 signature was associated with overall survival (OS) in the TCGA‐BRCA cohort (PanCancer Atlas 2018; *n* = 1082, 151 deaths). As a continuous predictor, the Mac‐SPP1 score was not significantly associated with OS (hazard ratio [HR] 0.94 per log2‐unit, 95% CI: 0.82–1.09, *p* = 0.43), and this remained non‐significant after adjustment for age, pathologic stage, and PAM50 subtype (adjusted HR 0.99, 95% CI: 0.85–1.14, *p* = 0.85). A median‐split Kaplan–Meier analysis likewise showed no significant difference (log‐rank *p* = 0.99, Figure 3B), with a median OS of 127.3 versus 129.6 months in the high‐ and low‐score groups, respectively. We therefore did not find evidence that the Mac‐SPP1 signature is prognostic in TCGA‐BRCA.

To investigate the underlying regulatory mechanisms, we analyzed TF activity across different macrophage subpopulations. Highly active TFs in Mac‐SPP1 included HOXA9, MAF, FOS, and JUND, which are associated with regulation of M2 macrophage polarization [24] (Figure 3C). We further assessed the metabolic profiles of these macrophages. Analysis of metabolic gene‐set enrichment score revealed that the Mac‐SPP1 cell subtype exhibited the highest metabolic gene‐set enrichment among the macrophage subtypes (Figure 3D); the Metastatic group also showed elevated metabolic enrichment relative to the Normal and Primary groups (Figure 3E), although this group comparison is subject to the dataset confound noted above. These findings characterize the Mac‐SPP1 macrophage subtype within the bone‐metastatic cohort.

### 3.4 Landscape, Functional Exhaustion, and Transcriptional Regulation of CD4+ T Cells in Bone Metastasis of Breast Cancer

After pooling T cells by identity, clustering analysis was performed to generate a UMAP that illustrates the intercellular phenotypic heterogeneity of each cell subpopulation (Figure 4A,B, Table S8). Nine subclusters were identified among T cells in different groups: CD8‐IL7R, CD4‐LEF1, CD8‐GZMK, T‐CTLA4‐CXCL13‐n, CD8‐GNLY, T‐CTLA4‐CXCL13‐P, CD4‐IKZF2, CD4‐S100A9, and CD8‐AFF3. The proportions of each cell type in all groups are shown in Figure 4C, and their marker genes are displayed in Figure 4E. Statistical analysis revealed that compared with the Primary group, the Metastatic group had significantly increased proportions of CD4‐IKZF2, CD4‐LEF1, CD4‐S100A9, CD8‐AFF3, CD8‐GNLY, and CD8‐GZMK (Figure 4D). As with the macrophage subtypes, these T‐cell subpopulations derive almost entirely from a single dataset (CD4‐S100A9, 257 of 262 cells; CD8‐GZMK, 2061 of 2065 cells, from the bone‐metastatic dataset), so these proportion differences are confounded with source dataset. Therefore, we first focused on the three CD4+ T cell subpopulations for further investigation. The CD4‐S100A9 subpopulation was annotated as T cells on the basis of robust co‐expression of the canonical T‐cell markers CD2, CD3D, and CD3E (Figure 5A). Although S100A9 is conventionally regarded as a myeloid marker, its expression can be induced in T cells under specific activation or stress conditions [25]. Consistent with this interpretation, CD4‐S100A9 cells displayed an activated yet exhausted transcriptional profile (high cytotoxicity and exhaustion scores) (Figure 4F and Table S9), suggesting that they represent a stressed CD4^+^ T‐cell state within the bone‐metastatic microenvironment rather than a distinct lineage or a misannotated myeloid population. Specifically, the Metastatic group showed a significantly higher cytotoxicity score compared with the Primary group and a significantly higher exhaustion score compared with the Normal group (Figure 4G). We conducted pseudotime analysis on CD4+ T cell subpopulations and identified three distinct cell states and a primary continuous trajectory (Figure 4H). Pseudotime analysis positioned CD4‐S100A9 cells at one end of the inferred trajectory and CD4‐LEF1 cells at the other. The CD4‐IKZF2 subpopulation was positioned at the intermediate stage of pseudotime, suggesting that this cluster represents a transitional or regulatory state during T cell differentiation.

**FIGURE 4:**
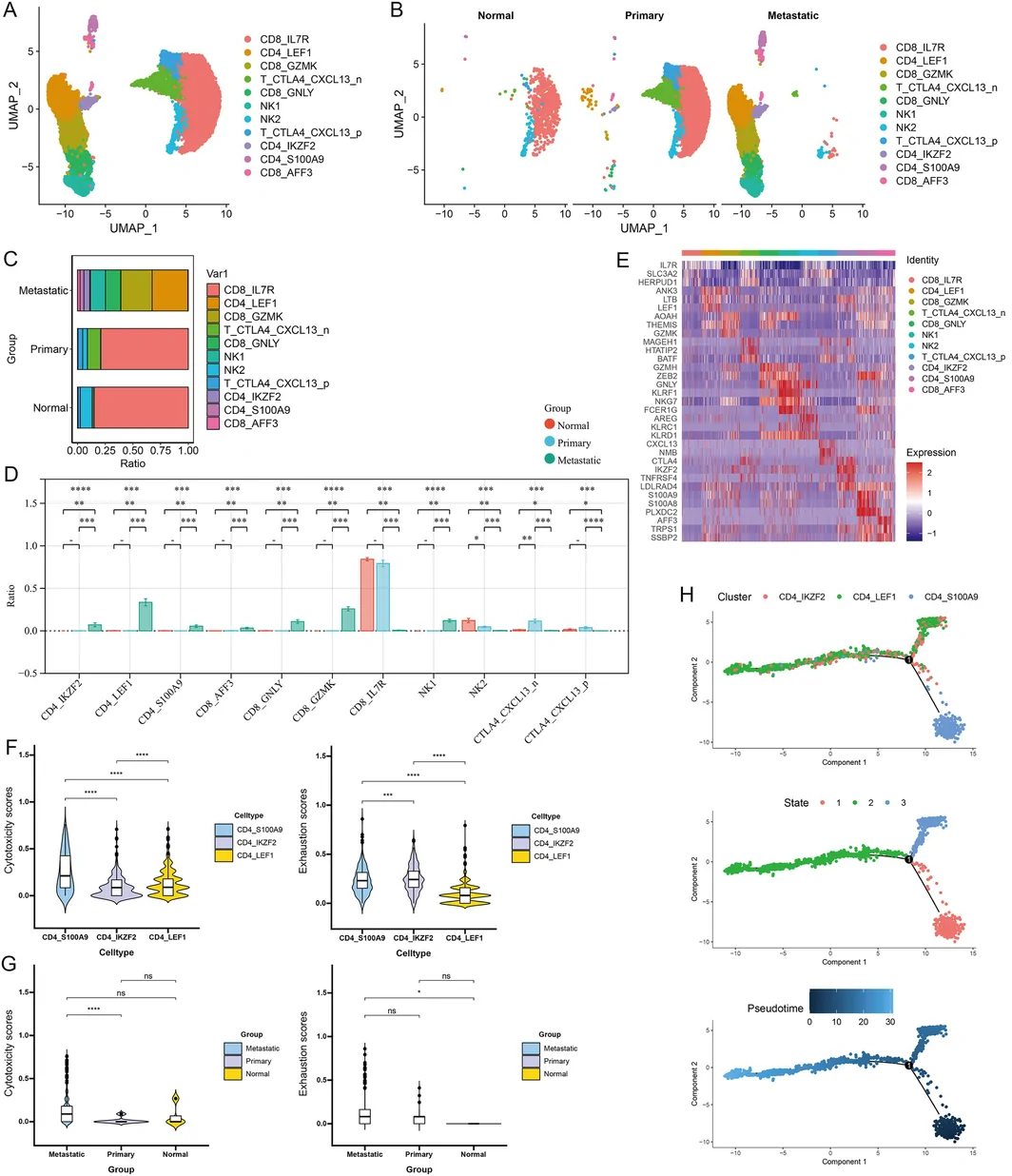
Transcriptional and cellular landscape of CD4+ T cells. (A, B) UMAP visualization of T cell subpopulations (A) in different groups (B). (C, D) Proportions of each T‐cell subpopulation among total T cells per group. (E) Heatmap showing expression levels of marker genes across different T cell subpopulations. (F, G) Cytotoxicity (GZMA, GZMB, GZMH, GZMK, GZMM, GNLY, PRF1, FASLG, IFNG, TNF, IL2R, IL2) and exhaustion (CXCL13, HAVCR2, PDCD1, TIGIT, LAG3, CTLA4, LAYN, RBPJ, VCAM1, GZMB, TOX, MYO7A) scores were computed per cell with the singscore algorithm. (H) Trajectory inference reveals a potential differentiation path among CD4^+^ T cell subpopulations.

**FIGURE 5:**
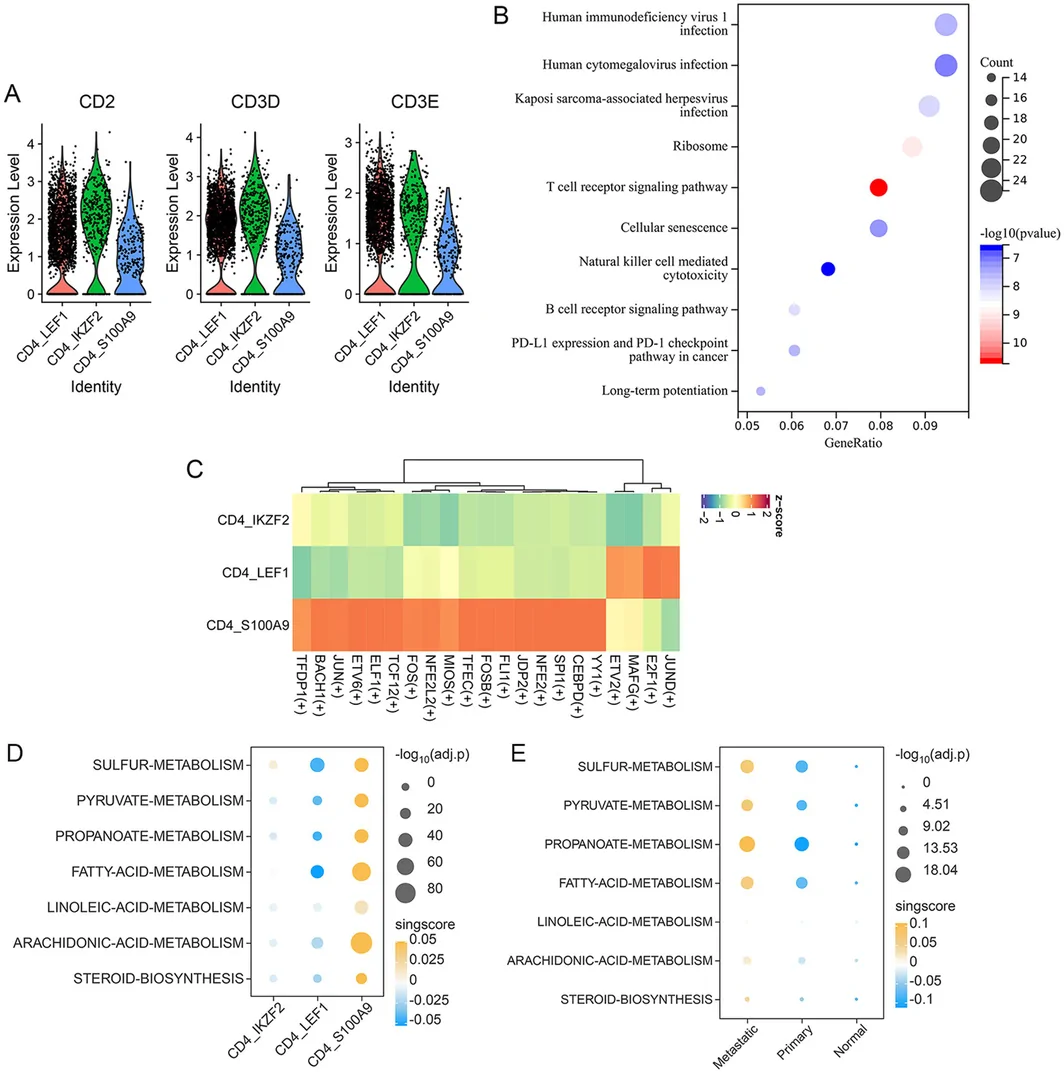
CD4^+^ T cell subpopulations undergo transcriptional, functional, and metabolic reprogramming. (A) The CD4‐S100A9 subpopulation was annotated as T cells on the basis of robust co‐expression of the canonical T‐cell markers CD2, CD3D, and CD3E. (B) Heatmap of TF activity signatures across CD4^+^ T subpopulations. (C) KEGG pathway enrichment analysis of DEGs in CD4^+^ T cells from the Primary and Metastatic groups. (D) Metabolic gene‐set enrichment score of the three CD4^+^ T cell subtypes. (E) Metabolic gene‐set enrichment score across the Normal, Primary, and Metastatic groups.

#### 3.4.1 CD4‐S100A9 T Cells in the Breast Cancer Bone‐Metastatic Microenvironment

TF activity analysis revealed that the CD4‐S100A9 subpopulation exhibited high activity of TFs such as YY1, CEBPD, SPI1, NFE2, JDP2, FLI1, FOSB, TFEC, MIOS, NFE2L2, FOS, TCF12, ELF1, ETV6, JUN, BACH1, and TFDP1, indicating its identity as an activated effector CD4+ T cell within a highly cytotoxicity and exhaustion stressed microenvironment (Figure 5B). KEGG analysis of the DEG profiles in CD4+ T cells of the Primary and Metastatic groups revealed strong associations with pathways such as the T cell receptor signaling pathway (Figure 5C). We then evaluated the metabolic characteristics of these CD4^+^ T cells. The CD4‐S100A9 subtype exhibited the highest metabolic gene‐set enrichment (Figure 5D). Moreover, the Metastatic group showed higher metabolic gene‐set enrichment than the Normal and Primary groups (Figure 5E), again subject to the dataset confound described above. These results characterize the CD4‐S100A9 T‐cell subtype within the bone‐metastatic cohort; whether it contributes to metastatic progression remains to be determined.

### 3.5 Functional Dynamics and Developmental Trajectory of CD8 ^+^ T Cells in the Tumor Microenvironment

We evaluated the levels of cytotoxicity and exhaustion of four CD8^+^ T cell subpopulations (CD8‐AFF3, CD8‐GNLY, CD8‐GZMK, and CD8‐IL7R), implying that CD8‐AFF3, CD8‐GNLY, and CD8‐GZMK had high cytotoxicity and exhaustion scores (Figure 6A and Table S10). Cytotoxic and exhausted CD8^+^ T cells tended to exhibit increased proportions in the Metastatic group (Figure 6B and Table S10). To further explore the dynamic transitional process of CD8^+^ T cells, we constructed a pseudotime map of CD8^+^ T cell state trajectory. To further explore the dynamic relationships among CD8^+^ T‐cell states, we constructed an inferred pseudotime trajectory (Figure 6C). Consistent with their low cytotoxicity and exhaustion scores, CD8‐IL7R cells, which display a central‐memory‐like phenotype, were positioned at the root of the trajectory. Along the inferred trajectory, cells progressed toward subsets with progressively higher effector and exhaustion programs. CD8‐GZMK cells occupied an intermediate position, retaining an effector program (GZMK) while beginning to acquire exhaustion‐associated features, suggesting a transition from an effector‐memory state toward terminal differentiation. The trajectory then resolved into two distinct terminal states: a cytotoxic terminal population (CD8‐GNLY) and an exhausted population (CD8‐AFF3). We note that pseudotime infers a transcriptional ordering rather than an established lineage relationship.

**FIGURE 6:**
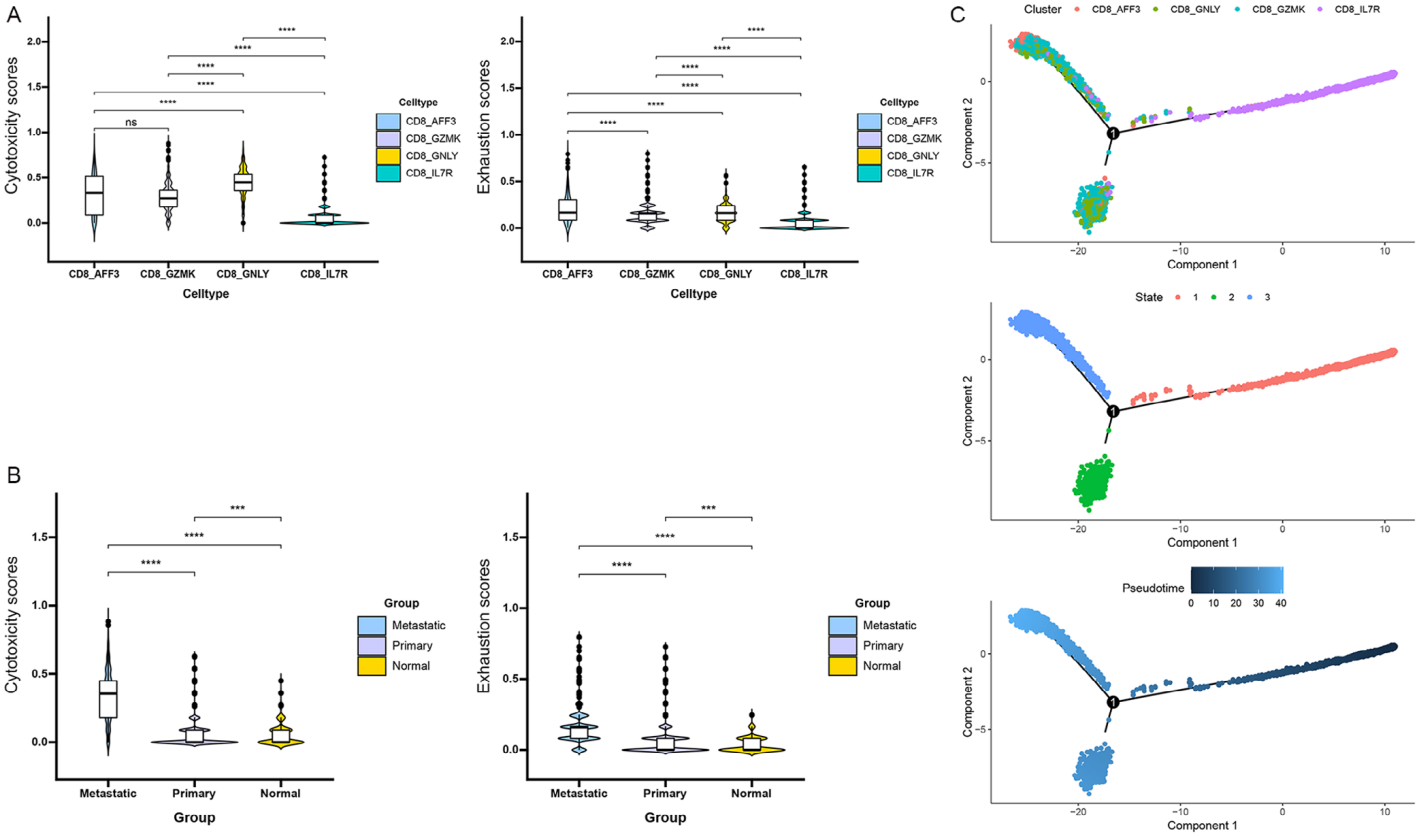
Cytotoxic and exhaustion phenotypes of CD8^+^ T cells and their differentiation trajectory. (A) Cytotoxicity and exhaustion scores across CD8^+^ T cell subpopulations and (B) their distribution in the Normal, Primary, and Metastatic groups. (C) Trajectory inference reveals a potential differentiation path among CD8^+^ T cell subpopulations.

### 3.6 Mac‐SPP1 Shows Predicted Communication With CD4‐S100A9 and CD8‐GZMK T Cells

TF activity analysis revealed that ETV1 is highly expressed in CD8‐GZMK cells (Figure 7A). KEGG pathway enrichment results indicate that DEGs between the Metastatic and Primary groups are primarily associated with ribosome, T cell receptor signaling pathway, and PD‐L1 expression and PD‐1 checkpoint pathway in cancer signaling pathways (Figure 7B). The metabolic characteristics of these CD8^+^ T cells were further evaluated. As shown in Figure 7C, all three effector CD8+ subsets (CD8‐AFF3, CD8‐GNLY, CD8‐GZMK) exhibited elevated metabolic gene‐set enrichment, with CD8‐AFF3 the highest, while CD8‐GZMK was comparable to CD8‐GNLY. The Metastatic group exhibited higher metabolic gene‐set enrichment than the Normal and Primary groups (Figure 7D), subject to the dataset confound described above. These findings characterize the CD8‐GZMK T‐cell subtype within the bone‐metastatic cohort.

**FIGURE 7:**
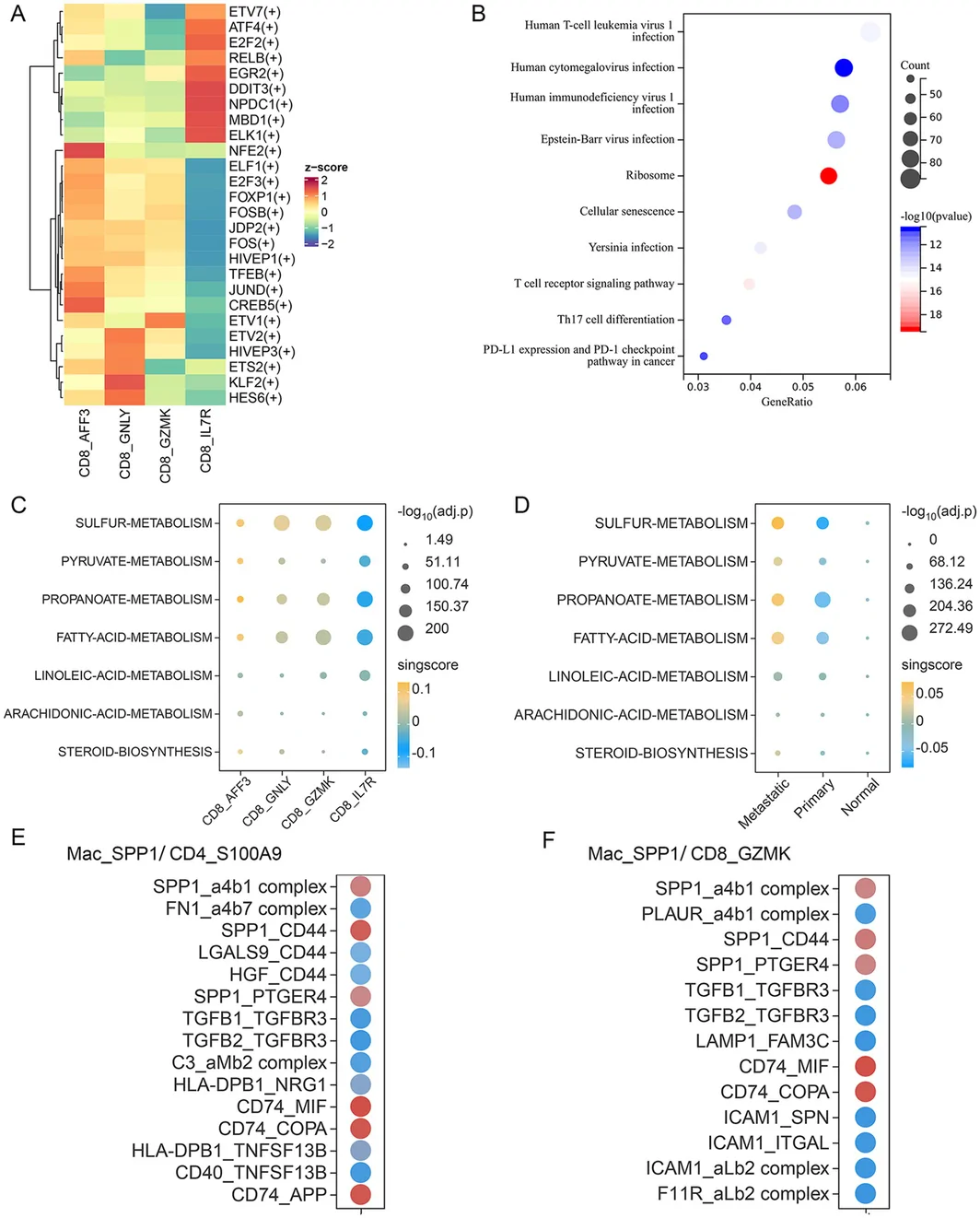
Transcriptional and cellular landscape of CD8^+^ T cells and predicted communication with Mac‐SPP1 macrophages. (A) Heatmap of TF activity signatures across CD8^+^ T subpopulations. (B) KEGG pathway enrichment analysis of DEGs in CD8^+^ T cells from the Primary and Metastatic groups. (C) Metabolic gene‐set enrichment score of the three CD8^+^ cell subtypes. (D) Metabolic gene‐set enrichment score across the Normal, Primary, and Metastatic groups. (E, F) Predicted cell–cell communication between Mac‐SPP1 and CD4‐S100A9 (E) and Mac‐SPP1 and CD8‐GZMK (F), inferred from ligand‐receptor co‐expression.

Cell–cell communication analysis revealed that the Mac‐SPP1 macrophage subpopulation may signal to distinct T cell subsets via specific ligand‐receptor pairs (Figure 7E,F, Tables S11 and S12). The interactions between Mac‐SPP1 and CD4‐S100A9/CD8‐GZMK T cells were predominantly mediated by SPP1‐related pathways (e.g., SPP1‐a4b1 and SPP1‐CD44), as well as immunomodulatory molecular pairs such as CD74‐MIF and CD74‐COPA.

## 4 Discussion

This study provides a single‐cell characterization of the breast cancer bone‐metastatic microenvironment, identifying an SPP1‐expressing macrophage subset (Mac‐SPP1) and predicting its interactions with CD4‐S100A9 and CD8‐GZMK T cells. We identified a Mac‐SPP1^+^ macrophage subset that was enriched in the metastatic group of our integrated cohort, exhibited M2 polarization, and showed elevated metabolic gene‐set enrichment, consistent with previous reports on SPP1^+^ TAMs in other cancers [26]. Previous studies have demonstrated that SPP1^+^ macrophages can regulate resistance to neoadjuvant chemotherapy and immunotherapy in esophageal squamous cell carcinoma [27] and promote the development and progression of head and neck squamous cell carcinoma via TNF‐α and IL‐1β secretion [28]. In colorectal cancer, SPP1^+^ macrophages interact with FAP^+^ fibroblasts to jointly promote tumor growth [29]. They also contribute to an immunosuppressive microenvironment through interactions with cancer‐associated fibroblasts, thereby facilitating colorectal cancer liver metastasis [30]. We note, however, that the Mac‐SPP1 signature was not significantly associated with overall survival in TCGA‐BRCA, so any prognostic value remains unestablished. The high activity of TFs such as HOXA9 and MAF suggests a regulatory network associated with an immunosuppressive, pro‐tumorigenic phenotype. Furthermore, the elevated metabolic gene‐set enrichment in Mac‐SPP1+ cells and the overall metastatic niche suggests a microenvironment enriched for metabolic programs that may support tumor survival and growth.

T cells represent a major immune cell population that plays a crucial role in improving clinical outcomes in various cancers, including breast and liver cancers [31, 32]. Among T cells, we identified a CD4‐S100A9+ subpopulation that was present almost exclusively in the bone‐metastatic dataset. This subset exhibited a unique transcriptional profile indicative of an activated yet exhausted effector state, as evidenced by high cytotoxicity/exhaustion scores and pronounced metabolic gene‐set enrichment. Its positioning at one end of the inferred trajectory suggests it represents an early, stress‐responsive CD4+ T cell state within the metastatic niche. CD8+ T cells, which are key members of the adaptive immune system, also play a definitive role in tumors [33]. In the present study, the CD8‐GZMK^+^ T cells showed features of exhaustion and high metabolic gene‐set enrichment, consistent with a dysfunctional yet metabolically active transcriptional state often observed in progressive tumors.

Evidence suggests that the interplay between macrophages and T cells can drive tumor progression [34]. For instance, Trehan et al. demonstrated an interaction between SPP1+ macrophages and tumor‐reactive CD8+ T cells in liver cancer cells [35]. Another study demonstrated the interaction between SPP1+ TAMs and CD8+ exhausted T cells in metastatic gastric cancer [36]. However, the role of macrophages and T cells in breast cancer bone metastasis has not been reported. In the present study, cell–cell communication analysis predicted crosstalk between Mac‐SPP1+ macrophages and T‐cell subsets, largely mediated by SPP1‐integrin and CD74‐related signaling pathways. These predictions suggest a potential mechanism by which macrophages may signal toward T‐cell dysfunction, potentially promoting exhaustion or altering differentiation, consistent with emerging evidence in glioblastoma [37]; this remains a computational prediction requiring experimental validation.

In summary, our single‐cell transcriptomic analyses of breast cancer bone metastasis reveal a coordinated interplay between specific macrophage and T cell subsets that is associated with an immunosuppressive, metabolically adapted microenvironment in the bone‐metastatic niche. Whether the Mac‐SPP1‐CD4‐S100A9/CD8‐GZMK interactions represent a therapeutically tractable axis for disrupting bone metastasis remains to be established experimentally. This study has several limitations that should be considered when interpreting our findings. First, the analyses relied entirely on publicly available single‐cell datasets, and the number of bone‐metastasis samples is limited. Second, primary tumors and bone metastases were derived from different datasets (GSE243526 and GSE266330, respectively), so biological condition is fully confounded with source dataset; the annotated macrophage and T‐cell subpopulations were themselves largely dataset‐specific, and we therefore interpret the study as a descriptive characterization of the bone‐metastatic cohort rather than a comparison across the normal‐primary‐metastasis trajectory. Third, the comparisons also contrast different tissue environments (bone versus breast), and no spatial information was available. Fourth, several patient characteristics, including age, menopausal status, molecular subtype, and treatment history, are incompletely reported in the source datasets and may influence the tumor microenvironment. Finally, as a purely computational reanalysis of observational transcriptomic data, this study offers associative rather than causal evidence. Confirming the functional roles of the Mac‐SPP1 and CD4‐S100A9/CD8‐GZMK subsets will require experimental validation (e.g., in vitro coculture and in vivo models), and larger, matched cohorts combined with spatial profiling will be needed to corroborate and extend these findings. In addition, because osteoclasts and osteoblasts were not resolved as separate clusters in our integrated dataset, the cell–cell communication analysis was limited to the immune subsets observed in the bone‐metastatic cohort; their crosstalk with Mac‐SPP1 macrophages thus remains to be addressed in future studies with protocols optimized for bone‐resident cell recovery.

## Author Contributions

**Zixue Xuan:** conceptualization, methodology. **Zhongxiu Wu:** methodology, writing – original draft. **Yuting Zhong:** methodology, software. **Jinying Jiang:** methodology, validation. **Yuan Zhang:** writing – original draft. **Lei Cheng:** methodology, formal analysis. **Jun Zhang:** formal analysis, writing – review and editing, funding acquisition. **Kai Wang:** visualization, writing – review and editing.

## Funding

This work was supported by Key Laboratory for Cancer Prevention and treatment of Guizhou Province (QKHPT[2025]031), Key Laboratory of Clinical Pharmacology and Rational Drug Use of Bijie City (BKHPT[2025]17), Zhejiang Administration of Traditional Chinese Medicine (GZY‐ZJ‐KJ‐23057), and the Bijie Science and Technology Joint Fund Project (BKLH[2025]71, BKLH[2023]63). Guizhou Provincial Basic Research Program in Natural Sciences: Youth Guidance Project (QN[2025]410). The funders had no role in study design; collection, analysis, and interpretation of data; writing of the report; or the decision to submit the report for publication.

## Ethics Statement

The authors have nothing to report.

## Consent

The authors have nothing to report.

## Conflicts of Interest

The authors declare no conflicts of interest.

## Supporting information


**Table S1:** Patient information for the samples analyzed in this study.
**Table S2:** Marker genes for the initial clusters identified by unsupervised clustering of the integrated single‐cell dataset.
**Table S3:** Marker genes for the major cell types.
**Table S4:** Proportions of each major cell type across the Normal, Primary, and Metastatic groups.
**Table S5:** Marker genes for the myeloid subpopulations.
**Table S6:** Marker genes for the macrophage subpopulations.
**Table S7:** M1 and M2 polarization scores (singscore) per cell for the macrophage subpopulations.
**Table S8:** Marker genes for the T‐cell subpopulations.
**Table S9:** Cytotoxicity and exhaustion scores (singscore) per cell for the CD4+ T‐cell subpopulations.
**Table S10:** Cytotoxicity and exhaustion scores (singscore) per cell for the CD8+ T‐cell subpopulations.
**Table S11:** Complete CellPhoneDB output for predicted ligand–receptor interactions between the Mac‐SPP1 macrophage subset and CD4+ T‐cell subpopulations.
**Table S12:** Complete CellPhoneDB output for predicted ligand–receptor interactions between the Mac‐SPP1 macrophage subset and CD8+ T‐cell subpopulations.
**Table S13:** Effect sizes (Cliff's delta) with 95% confidence intervals and *p* values for all cell‐type proportion comparisons across groups.

## Data Availability

The datasets used and/or analyzed during the current study are available from public repositories: scRNA‐seq data were obtained from GSE266330 and GSE243526, which were downloaded from the GEO database (https://www.ncbi.nlm.nih.gov/geo/). The transcriptome and clinical data were retrieved from TCGA‐BRCA cohort (https://www.cancer.gov/tcga). Pathway enrichment analysis was performed using the KEGG database (https://www.kegg.jp/kegg/pathway). Ligand‐receptor interaction analysis was conducted using CellPhoneDB v5.0 (https://www.cellphonedb.org). The data that supports the findings of this study are available in the Supporting Information of this article. A preprint version of this manuscript has been posted on Research Square: “SPP1+ Macrophage‐T Cell Axis Orchestrates the Immune Microenvironment in Breast Cancer Bone Metastasis” (DOI: 10.21203/rs.3.rs‐8805630; https://www.researchsquare.com/article/rs‐8805630/v1). This manuscript is a revised version of the same study submitted to this journal.
